# A Novel Murine Model of Parvovirus Associated Dilated Cardiomyopathy Induced by Immunization with VP1-Unique Region of Parvovirus B19

**DOI:** 10.1155/2016/1627184

**Published:** 2016-10-12

**Authors:** Julijus Bogomolovas, Egidijus Šimoliūnas, Ieva Rinkūnaitė, Luka Smalinskaitė, Andrej Podkopajev, Daiva Bironaitė, Cleo-Aron Weis, Alexander Marx, Virginija Bukelskienė, Norbert Gretz, Virginija Grabauskienė, Dittmar Labeit, Siegfried Labeit

**Affiliations:** ^1^Department of Integrative Pathophysiology, Medical Faculty Mannheim, Mannheim, Germany; ^2^Department of Pathology, Forensic Medicine and Pharmacology, Faculty of Medicine, Vilnius University, Vilnius, Lithuania; ^3^Department of Biological Models, Institute of Biochemistry, Vilnius University, Vilnius, Lithuania; ^4^State Research Institute, Centre for Innovative Medicine, Department of Stem Cell Biology, Vilnius, Lithuania; ^5^Medical Faculty Mannheim, Institute of Pathology, Mannheim, Germany; ^6^Medical Faculty Mannheim, Medical Research Centre, Mannheim, Germany

## Abstract

*Background*. Parvovirus B19 (B19V) is a common finding in endomyocardial biopsy specimens from myocarditis and dilated cardiomyopathy patients. However, current understanding of how B19V is contributing to cardiac damage is rather limited due to the lack of appropriate mice models. In this work we demonstrate that immunization of BALB/c mice with the major immunogenic determinant of B19V located in the unique sequence of capsid protein VP1 (VP1u) is an adequate model to study B19V associated heart damage.* Methods and Results*. We immunized mice in the experimental group with recombinant VP1u; immunization with cardiac myosin derived peptide served as a positive reference and phosphate buffered saline served as negative control. Cardiac function and dimensions were followed echocardiographically 69 days after immunization. Progressive dilatation of left ventricle and decline of ejection fraction were observed in VP1u- and myosin-immunized mice. Histologically, severe cardiac fibrosis and accumulation of heart failure cells in lungs were observed 69 days after immunization. Transcriptomic profiling revealed ongoing cardiac remodeling and immune process in VP1u- and myosin-immunized mice.* Conclusions*. Immunization of BALB/c mice with VP1u induces dilated cardiomyopathy in BALB/c mice and it could be used as a model to study clinically relevant B19V associated cardiac damage.

## 1. Introduction

Dilated cardiomyopathy (DCM) remains the leading cause of heart failure leading to heart transplantation. When therapeutic and prophylactic measures are applied early, a progression of ischemic cardiomyopathy can be stopped and patient can be saved from the heart transplantation. However, only in selected cases progression of primary nonischemic dilated cardiomyopathy can be circumvented with the currently available therapy. Thus, a high demand for new DCM therapies exists. Improved diagnostics revealed that up to 70% of primary nonischemic DCM cases might be due to an unfortunate combination of immunological factors and viral agents [1].

Numerous reports on the presence of parvovirus B19 (B19V) in the hearts of DCM patients imply that B19V might be causatively linked to the heart damage, but the understanding of its pathogenicity in heart diseases is rather limited [2]. B19V infection in humans typically manifests as* erythema infectiosum*, hydrops fetalis in pregnant women or aplastic crisis, infecting and replicating in erythroid progenitor cells. Up to 60% of adult population are positive for B19V IgG antibodies indicating a previous infection [3]. Following the B19V infection, a dominant immune response is elicited by the VP1-unique region (VP1u), which harbors potent neutralizing epitopes [4, 5]. VP1u is an N-terminal 227 amino acid long region distinguishing minor capsid component VP1 from the major VP2 protein. VP1u region possesses phospholipase activity critical for infectivity [6] and acts as a major determinant of viral tropism [7]. Upon cardiac B19V infection, viral genomes are detected only in endothelial cells and not in myocytes [2]. Therefore, it is very likely that mechanisms beyond the viral infection are involved in the pathogenesis of B19 related DCM. It has been proposed that viral infection can trigger pathological immune response leading to the development of DCM [1].

So far two studies reported on cardiac response to VP1u immunization. Active immunization of BALB/c mice with VP1u induced cardiac inflammation and increased blood levels of nonspecific cytosolic enzymes [8]. Passive immunization of ZB/W F1 mice with rabbit anti-B19-VP1u IgG elicited foci of cardiac infiltration and upregulation of cardiac matrix metalloproteinase-9 (MMP9) activity. However, no significant differences in the cardiac atrial natriuretic peptide, brain natriuretic peptide, heart-type fatty acid-binding protein, and creatine kinase MB levels were detected among all experimental groups [9]. Nevertheless, cardiac function was not evaluated in these studies and progression/resolution of cardiac damage was not followed in these studies. Thus, in this work we demonstrate that VP1u holds cardiopathic potential in the experimental paradigm of widely accepted myosin-induced myocarditis model. Cardiac damage is induced by immunization of BALB/c mice with a cardiac myosin derived peptide. Immunized animals developed acute myocarditis accompanied inflammatory infiltrates about 21 days after immunization and subsequent progression to DCM marked by cardiac fibrosis and impaired systolic function around 40 to 70 days after immunization [10]. To our knowledge, this is the first murine model to study B19V induced DCM.

## 2. Materials and Methods

### 2.1. Animal Experiments

Experiments were performed on 6–8-week-old BALB/c male mice. Animals were housed in standard plastic cages in an animal room with controlled environmental conditions and maintained on standard food and water* ad libitum*. Experimental procedures conformed to Directive 2010/63/EU requirements and were approved by the Lithuanian State Food and Veterinary Service (approval number G2-17, 2014/11/11).

### 2.2. Immunization Protocol

Mice were immunized as described [10]. A peptide derived from *α*-myosin (MyHC-*α*614-62 RSLKLMATLFSTYASADR) or recombinant VP1u (residues 3–229, UniProtKB/Swiss-Prot: P07299.1) produced in endotoxin-deficient ClearColi (Lucigen)* E. coli* strain as described earlier [11] was used for immunization. 150 *μ*g/animal of cardiac myosin or 500 *μ*g/animal of VP1u was used per immunization. Animals were immunized twice in a 7-day interval. Peptide and protein dissolved in PBS were mixed with equal amount of Freund's complete adjuvant for the first and incomplete Freud's adjuvant for second immunization, emulsified using sonifier, and injected subcutaneously. During the first immunization, animals were stimulated with 500 ng intraperitoneal injection of* Pertussis* toxin in bovine serum albumin supplemented sterile saline. Control animals were immunized using PBS mixed with Freund's adjuvant following the same protocol described above.

### 2.3. Echocardiography

Transthoracic M-mode echocardiography was performed under isoflurane (1% in pure oxygen) anesthesia, using linear 13 MHz sensor coupled to HITACHI EUB-7000HV ultrasound system. The heart was viewed in short parasternal axis at the papillary muscle level. Six cardiac cycles per measurement were used for averaging. Animals were examined on the day of the first immunization and then 20, 47, and within 60–69 days later. LV ejection fraction was calculated using Teichholz method from M-mode images [12].

### 2.4. Histology

60 to 69 days after immunization, animals were sacrificed and heart and lungs were fixed in 10% neutral buffered formalin, embedded in paraffin, sectioned at 6 *μ*m, and stained with picrosirius or Prussian blue (ScyTek) following manufacturer's recommendations. The degree of fibrosis was graded by an experienced pathologist on picrosirius stained sections under white light; density of alveolar siderophages in lung parenchyma was quantified using semiautomated routine in Fiji [13]. For CD45 immunohistochemistry, reagents from Dako Ltd. were used. Heat-mediated antigen retrieval was performed. Dakoreal™ EN Vision TM Detection System Peroxidase/DAB was used to visualize the signal. Rabbit polyclonal anti-CD45 antibody (Abcam ab10558) was used at 1 : 1000 dilution. CD45 positive foci were quantified by counting in 10 random fields (magnification 400x).

### 2.5. Expression Profiling

Total RNA was extracted from frozen apical LV region using TRIzol® Plus RNA Purification Kit (ThermoFisher Scientific). Gene expression profiling with Affymetrix system using GeneChip® Mouse Gene 2.0 ST Arrays was performed in Microarray Core Facility at Medical Research Center. Arrays were processed using software package JMP® Genomics. Gene set enrichment analysis was performed using GSEA-P [14] with KEGG pathway database derived gene sets [15]. Gene sets with *p* value and *q*-val FDR < 0.05 were considered significantly dysregulated and visualized with Enrichment Map [16].

### 2.6. Statistics

Changes in echocardiographic parameters were statistically evaluated using mixed ANOVA. Differences between fibrosis scores and alveolar siderophage densities were statistically assessed using The Kruskal-Wallis *H* test. The analysis was performed using SPSS 23; *p* values < 0.05 were considered significant.

## 3. Results

### 3.1. Immunization with VP1u Causes Progressive Worsening of Systolic Function in BALB/c Mice

We have followed heart morphology and cardiac function in mice by echocardiography over the course of 69 days after immunization (pi). Given timeframe encloses acute myocarditis (21 days pi) and DCM (60 days pi) phases reported for the established myosin-induced myocarditis/DCM model [17, 18].

**Figure 1:**
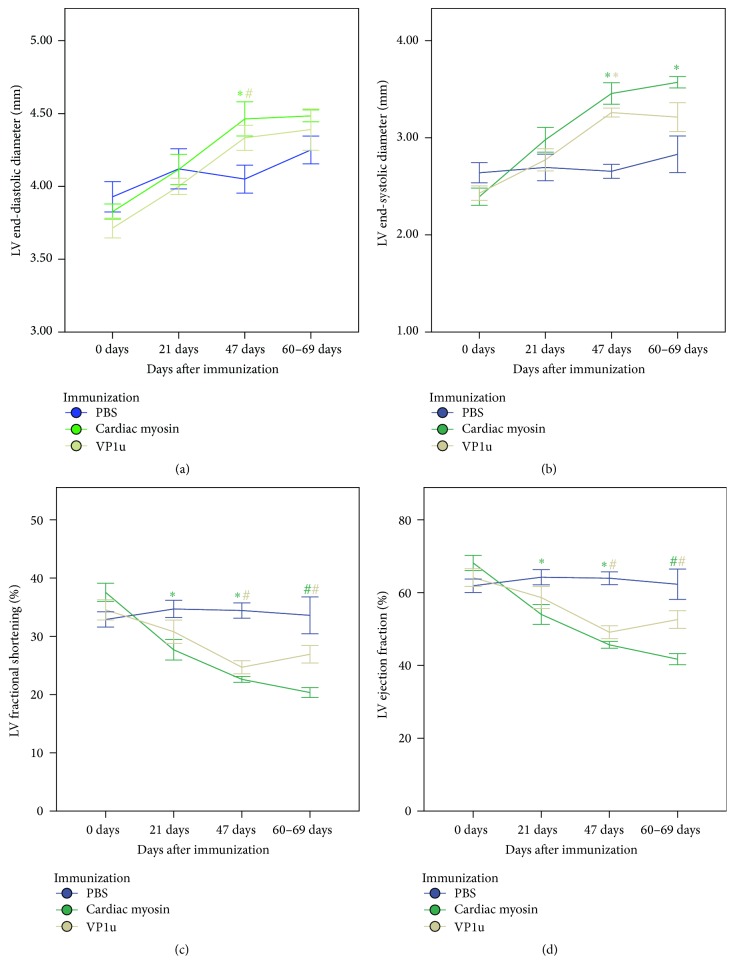
Serial echocardiographic measurements of immunized animals. All parameters were determined from serial echocardiography images obtained at given intervals in isoflurane-anesthetized mice. LV diameters were measured in M-mode at the short parasternal axis. Data are expressed as means ± SEM; *n* = 6–10; *∗* denotes *p* < 0.005; # denotes *p* < 0.05, from 0 days.

There was a statistically significant effect of the antigen on echocardiographic parameters in time-dependent manner. Immunizations with cardiac myosin and VP1u, but not with PBS, lead to statistically significant changes in LV end-diastolic and end-systolic diameters, fractional shortening, and LV ejection fraction over time (Table 1). Immunization with cardiac myosin and VP1u caused progressive dilatation of LV, resulting in gradual increase of LV diameter at systole and diastole (Figure 1). In good agreement with DCM phenotype, remodeling of LV was accompanied by the worsening of systolic function, measured by fractional shortening and ejection fraction. Immunization with cardiac myosin and VP1u resulted in statistically significant decrease of EF and FS as early as 20 days pi (Figure 1).

### 3.2. Immunization with VP1u Leads to Cardiac Fibrosis

**Figure 2:**
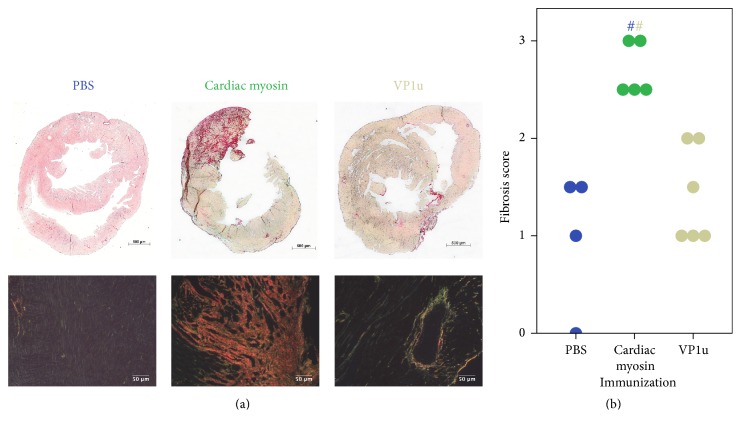
Fibrotic changes in the myocardium. Representative fields for each group are shown. Sections are photographed under white light (fibrosis-red, cardiac tissue-yellow to pink, upper panel) and under polarized light (thin collagen fibers are green, thick-red; the background is black, lower column). Distribution of fibrosis scores is given in (b). # denotes *p* < 0.05; color marks the compared group, *n* = 4–6.

69 days after immunization hearts and lungs were examined histologically. Cardiac sections were stained with picrosirius red to visualize fibrosis and imaged under the white and polarized light (Figure 2). In cardiac sections of PBS-immunized mice no to mild fibrosis could be observed, evenly distributed through myocardium, physiologically more intensive around the vessels. Under polarized light, mostly green birefringence was observed, typical for thin collagen fibers. Myosin-immunized mice developed severe fibrosis clustering into multiple foci, mostly located in the pericardium, consisting of thick (red) and thin (green) collagen fibers. In VP1u animals, fibrotic foci developed around the vessels, mimicking pathological picture of “hypertensive” cardiomyopathy. Moreover, under the polarized light, extensive spread of interstitial fibrosis could be seen. The extent of cardiac fibrosis was graded by an experienced pathologist in a blinded manner. A Kruskal-Wallis *H* test confirmed significantly different median fibrosis scores between studied groups,* H*(2) = 9.987, *p* = 0.007. Pairwise comparisons were performed using Dunn's procedure with a Bonferroni correction for multiple comparisons. Adjusted *p* values are presented. This* post hoc* analysis revealed statistically significant differences in median fibrosis scores between the PBS (4.62) and cardiac myosin (13) (*p* = 0.013) and VP1u- (6.08) and cardiac myosin-immunized animals (*p* = 0.028), but not between VP1u- (5.50) and PBS-immunized animals.

### 3.3. Accumulation of Heart Failure Cells in Lungs of VP1u-Immunized Animals

**Figure 3:**
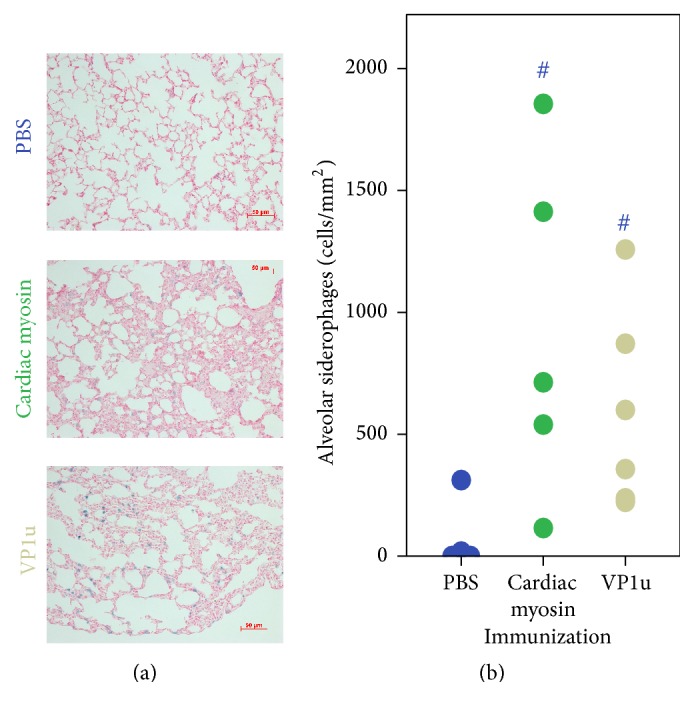
Accumulation of alveolar siderophages, indicative of heart failure in lungs of cardiac myosin- or VP1u-immunized animals. Representative fields are shown for each group. Siderophages stain blue in red counterstained lung tissue. Distribution of median alveolar siderophage densities is given in (b); # denotes *p* < 0.05; color marks the compared group, *n* = 4–6.

We have stained lungs of animals with Prussian blue for the visualization of siderophages known as the heart failure cells (Figure 3). Upon left heart failure, increased pulmonary blood pressure causes red blood cells to leak out, where they are engulfed by macrophages. The resulting accumulation of hemosiderin in macrophages stains blue. Cardiac myosin-immunized animals displayed the accumulation of alveolar siderophages compared to physiological levels observed in lungs of PBS-immunized animals. A Kruskal-Wallis *H* test confirmed significantly different median siderophage densities between studied groups,* H*(2) = 6.514, *p* = 0.039. Pairwise comparisons were performed using Dunn's procedure with a Bonferroni correction for multiple comparisons. Adjusted *p* values are presented. This* post hoc* analysis revealed statistically significant differences in median fibrosis scores between the PBS (3.25) and cardiac myosin (10.6) (*p* = 0.043) and between VP1u- (9.0) and PBS-immunized animals (*p* = 0.049).

### 3.4. Transcriptomic Profiling of Immunized Animals

**Figure 4:**
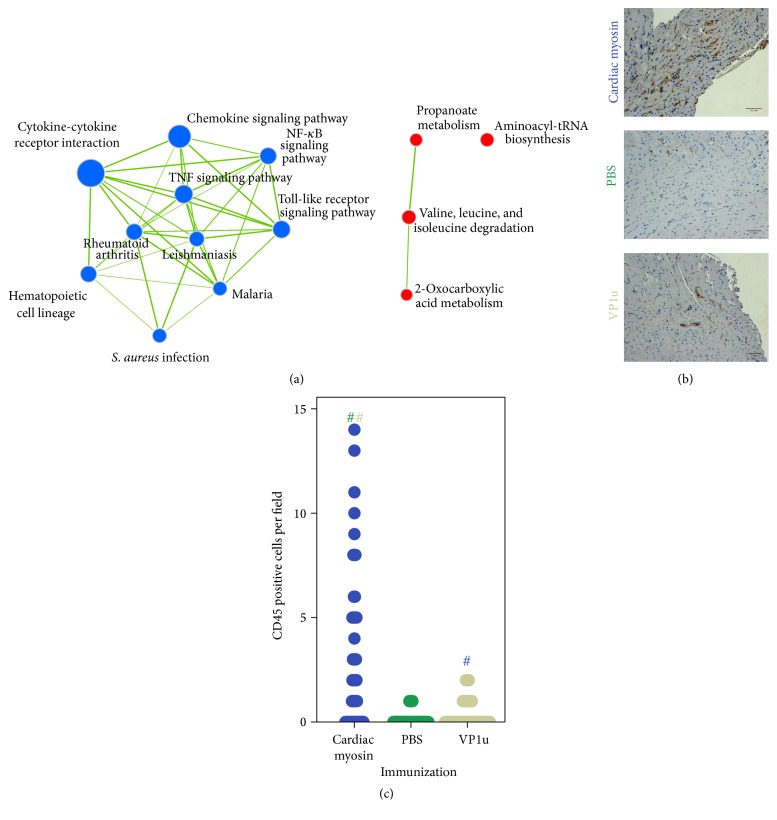
Immune infiltration in immunized animals. Network representation of under- and overrepresented gene sets between cardiac myosin- and VP1u-immunized animals (a). Gene sets related to immunity and infection are underrepresented in VP1u-immunized animals (blue nodes), whereas gene sets involved in catabolic and anabolic processes are overrepresented (red nodes). Accumulation of CD45 positive foci in cardiac myosin-immunized animals (b). Number of CD45 positive cells is given in (c); # denotes *p* < 0.05; color marks the compared group.

To gain molecular insights into VP1u immunization triggered heart damage we have performed gene expression profiling on left ventricular samples. 362 genes were found statistically significantly dysregulated between studied groups (Supplementary Table  1 in Supplementary Material available online at http://dx.doi.org/10.1155/2016/1627184). The list of top 10 dysregulated genes (Table 2) demonstrated shared and unique molecular features between myosin and VP1u triggered cardiac damage. In both animal models (compared to PBS control) matrix metallopeptidase 3 was upregulated, and tissue inhibitor of metalloprotease 4 was downregulated. This expression pattern resembles changes in the proteome, marking cardiac remodeling typical for the DCM [19, 20]. Moreover, commonly upregulated set of inflammation-related genes (*Igkc*,* Ighm*, and* Tcrg-C4*) imply activation of the immune system in the myocardium. However, studied models share some differences in gene expression patterns. Immunization with the VP1u results in less pronounced immune activation of the myocardium, when compared to myosin-immunized mice, resulting in downregulation of immunity-related genes (*Igkc, Igj, Trbj1-1, Mcpt4, and Mgl2*). The cellular basis of this phenomenon could be related to a significant accumulation of CD45 positive immune cells in hearts of myosin-immunized animals (Figure 4). Set of genes involved in central energy metabolism (*Pdk4, Gpam, and Glul*) was upregulated in VP1u-immunized animals. Speculatively, this could indicate a relatively spared energy metabolism in a less damaged myocardium of VP1u-immunized animals; on another hand this might mean differing molecular mechanisms leading to heart damage. Gene set enrichment analysis using KEGG annotation based gene set exemplifies differences between expression patterns of myosin- and VP1u-immunized animals. Gene sets related to inflammation and immune response are underrepresented in VP1u-immunized animals, whereas gene sets involved in energy metabolism are overrepresented.

## 4. Discussion

Echocardiographic and histological assessment of cardiac function and structure in VP1u-immunized animals revealed gradual remodeling of LV and worsening of systolic function. Observed changes are typical for DCM phenotype and follow the pattern of heart damage caused by established myosin-induced myocarditis/DCM model, used in this work as a positive reference. Thus immunization with VP1u can be applied as a model to study clinically relevant B19V associated DCM.

Our findings are in good agreement with previous studies, demonstrating that active or passive immunization of susceptible mice strain with VP1u alone can elicit heart damage [8, 9]. However, in this study we demonstrated that immunization with VP1u could progress beyond the phase of acute cardiac inflammation reported by Nie et al. and lead to LV dilatation, impaired systolic function, and cardiac fibrosis consistent with the development of DCM. This pattern closely resembles progression of heart damage elicited by the immunization with cardiac myosin derived peptide used in the established experimental autoimmune myocarditis/DCM model [10, 17].

However, cardiac damage in VP1u-immunized animals has unique features when compared to the cardiac myosin-immunized group. Animals in the VP1u-immunized group developed milder cardiac damage with less LV dilatation, lesser cardiac fibrosis, and fewer heart failure cells in lungs. Moreover, slight LV reverse remodeling was observed in VP1u-immunized animals 47 to 60–69 days after immunization. It is noteworthy that spontaneous recovery of cardiac function is well documented in clinical practice [21] and presented model could at least partly be used to study this relevant phenomenon.

At the molecular level, comparative transcriptomic profiling revealed that immunization with VP1u resulted in less pronounced upregulation of inflammation-related genes than immunization with cardiac myosin. Conversely, immunization with VP1u upregulated set of energy metabolism genes. Speculatively, observed differences in gene expression patterns might reflect the varying severity of heart damage caused by the immunization with distinct antigens. Significant accumulation of CD45 positive immune cells in hearts of the cardiac myosin-immunized animals supports this observation. However, these differences might also imply differing molecular mechanisms leading to the heart damage.

Intriguingly, cardiac fibrosis in VP1u animals clustered around the vessels and resembled the histological picture of hypertrophic cardiomyopathy. It was shown that B19V myocarditis is an endothelial cell-mediated disease [22, 23] and anti-B19V antibodies alone can activate endothelial cells* in vitro* [24]. Thus, endothelium of cardiac vessels might be a primary autoimmune target in VP1u-immunized animals.

In conclusion, presented data indicate that immunization of BALB/c mice with the recombinant VP1u protein causes heart damage compatible with the DCM phenotype. Described model can be useful to study clinically relevant B19V associated heart damage.

## Supplementary Material

List of dysregulated genes

## Figures and Tables

**Table 1:** Echocardiographic parameters of immunized animals.

Antigen	Days after immunization	LV end-diastolic diameter (mm)	LV end-systolic diameter (mm)	Fractional shortening (%)	LV ejection fraction (%)
		Mean ± SE	Mean ± SE	Mean ± SE	Mean ± SE
PBS	0	3.93 ± 0.10	2.64 ± 0.10	32.9 ± 1.3	61.9 ± 1.8
	20	4.12 ± 0.14	2.70 ± 0.14	34.7 ± 1.5	64.2 ± 2.1
	47	4.05 ± 0.10	2.66 ± 0.07	34.4 ± 1.3	64.0 ± 1.8
	60–69	4.25 ± 0.10	2.83 ± 0.19	33.6 ± 3.2	62.3 ± 4.2

*Simple main effect for measurement time*		*F(3, 9) = 1.025, p = 0.426, partial η* ^2^ * = 0.255*	*F(3, 9) = 0.574, p = 0.646, partial η* ^2^ * = 0.161*	*F(3, 9) = 1.010, p = 0.432, partial η* ^2^ * = 0.252*	*F(3, 9) = 1.060, p = 0.413* , *partial η* ^2^ * = 0.261*

Cardiac myosin	0	3.83 ± 0.05	2.39 ± 0.09	37.5 ± 1.6	68.1 ± 2.1
	20	4.12 ± 0.10	2.98 ± 0.13	27.7 ± 1.8	54.0 ± 2.7
	47	4.46 ± 0.12	3.46 ± 0.11	22.6 ± 0.5	45.7 ± 0.9
	60–69	4.48 ± 0.04	3.57 ± 0.06	20.4 ± 0.9	41.7 ± 1.5

*Simple main effect for measurement time*		*F(3, 12) = 25.761, p < 0.0005, partial η* ^2^ * = 0.866*	*F(3, 12) = 37.638, p < 0.0005, partial η* ^2^ * = 0.904*	*F(3, 12) = 35.975, p < 0.0005, partial η* ^2^ * = 0.900*	*F(3, 12) = 36.057, p < 0.0005, partial η* ^2^ * = 0.900*

VP1u	0	3.71 ± 0.07	2.43 ± 0.07	34.5 ± 1.7	64.1 ± 2.4
	20	4.00 ± 0.06	2.77 ± 0.11	30.8 ± 20	58.7 ± 3.0
	47	4.33 ± 0.09	3.26 ± 0.05	24.7 ± 1.1	49.1 ± 1.8
	60–69	4.39 ± 0.14	3.21 ± 0.15	26.9 ± 1.5	52.6 ± 2.4

*Simple main effect for measurement time*		*F(3, 15) = 9.760, p < 0.005, partial η* ^2^ * = 0.661*	*F(3, 15) = 18.526, p < 0.0005, partial η* ^2^ * = 0.787*	*F(3, 15) = 13.968, p < 0.0005, partial η* ^2^ * = 0.736*	*F(3, 15) = 13.707, p < 0.0005, partial η* ^2^ * = 0.733*

*Two-way interaction between immunization and the time on echocardiographic parameter*		*F(6, 36) = 3.755, p < 0.005, partial η* ^2^ * = 0.385*	*F(6, 36) = 8.669, p < 0.0005, partial η* ^2^ * = 0.591*	*F(6, 36) = 9.225, p < 0.0005, partial η* ^2^ * = 0.606*	*F(6, 36) = 9.460, p < 0.0005, partial η* ^2^ * = 0.612*

**Table 2:** Top 10 dysregulated transcripts.

		Fold change	*p* value			Fold change	*p* value
PBS *versus *cardiac myosin							

Igkc	Immunoglobulin kappa constant	−2.47	0.04	Timp4	Tissue inhibitor of metalloproteinase 4	2.94	0.01
Tfrc	Transferrin receptor	−1.80	0.01	Pdk4	Pyruvate dehydrogenase kinase, isoenzyme	2.65	0.02
Tcrg-C4	T cell receptor gamma, constant 4	−1.68	0.04	Fkbp5	FK506 binding protein 5	2.49	0.02
Mmp3	Matrix metallopeptidase 3	−1.45	0.05	Zbtb16	Zinc finger and BTB domain containing 16	2.09	0.02
Comp	Cartilage oligomeric matrix protein	−1.36	0.05	Fam107a	Family with sequence similarity 107, mem	2.06	0.02
Rrad	Ras-related associated with diabetes	−1.33	0.02	Map3k6	Mitogen-activated protein kinase kinase	2.04	0.02
Ighm	Immunoglobulin heavy constant mu	−1.31	0.02	Ucp3	Uncoupling protein 3 (mitochondrial, pro	1.97	0.03
Gpr22	G protein-coupled receptor 22	−1.06	0.01	Cdkn1a	Cyclin-dependent kinase inhibitor 1A (p21)	1.73	0.05
Map2k6	Mitogen-activated protein kinase kinase	−1.04	0.03	Ms4a4a	Membrane-spanning 4-domain, subfamily A	1.51	0.02
Igkj5	Immunoglobulin kappa joining 5	−1.04	0.04	Klhl38	Kelch-like 38	1.45	0.04

PBS *versus* VP1u							

Rrad	Ras-related associated with diabetes	−1.69	0.01	Fkbp5	FK506 binding protein 5	2.39	0.02
Kcnj2	Potassium inwardly-rectifying channel	−1.05	0.01	Timp4	Tissue inhibitor of metalloproteinase 4	2.04	0.03
Arhgap18	Rho GTPase activating protein 18	−1.01	0.01	Cdkn1a	Cyclin-dependent kinase inhibitor 1A (p21)	2.02	0.03
Ighm	Immunoglobulin heavy constant mu	−0.88	0.05	Map3k6	Mitogen-activated protein kinase kinase	2.01	0.02
Lphn2	Latrophilin 2	−0.86	0.03	Fam107a	Family with sequence similarity 107, mem	2.00	0.03
Tfrc	Transferrin receptor	−0.82	0.02	Serpine1	Serine (or cysteine) peptidase inhibitor	1.72	0.01
Dusp18	Dual specificity phosphatase 18	−0.71	0.03	Zbtb16	Zinc finger and BTB domain containing 16	1.71	0.03
Slc26a10	Solute carrier family 26, member 10	−0.68	0.01	8430408G22Rik	RIKEN cDNA 8430408G22 gene	1.69	0.02
Slc6a6	Solute carrier family 6 (neurotransmitter transporter, taurine), member 6	−0.68	0.04	Ms4a4a	Membrane-spanning 4-domain, subfamily A	1.60	0.02
Kctd12b	Potassium channel tetramerisation domain	−0.66	0.03	Ctgf	Connective tissue growth factor	1.32	0.01

Cardiac myosin *versus* VP1u							

Pdk4	Pyruvate dehydrogenase kinase, isoenzyme	−1.86	0.04	Igkc	Immunoglobulin kappa constant	2.54	0.03
Ces1d	Carboxylesterase 1D	−1.27	0.03	Trbj1-1	T cell receptor beta joining 1-1	1.73	0.04
Apln	Apelin	−0.96	0.05	Igj	Immunoglobulin joining chain	1.56	0.04
Ttn	Titin	−0.96	0.04	Comp	Cartilage oligomeric matrix protein	1.38	0.04
Klf15	Kruppel-like factor 15	−0.93	0.04	Mmp3	Matrix metallopeptidase 3	1.34	0.05
Ctnnal1	Catenin (cadherin associated protein), a	−0.93	0.01	Nox4	NADPH oxidase 4	1.07	0.04
Tacc2	Transforming Acidic Coiled-Coil Containing Protein 2	−0.84	0.02	Mcpt4	Mast cell protease 4	1.03	0.04
Scn4a	Sodium channel, voltage-gated, type IV	−0.82	0.03	Tfrc	Transferrin receptor	0.98	0.01
Gpam	Glycerol-3-phosphate acyltransferase, mi	−0.74	0.03	Rab2b	RAB2B, member RAS oncogene family	0.94	0.04
Cited2	Cbp/p300-interacting transactivator, wit	−0.73	0.04	Snord71	Small nucleolar RNA, C/D box 71	0.93	0.04
Fbxo32	F-box protein 32	−0.73145	0.04	Mgl2	Macrophage galactose N-acetyl-galactosamine specific lectin 2	0.91	0.01
Glul	Glutamate-ammonia ligase (glutamine synthase)	−0.68506	0.01	Glipr1	GLI pathogenesis-related 1 (glioma)	0.84	0.04
